# Rechargeable aluminum batteries: effects of cations in ionic liquid electrolytes†

**DOI:** 10.1039/c9ra00765b

**Published:** 2019-04-11

**Authors:** Guanzhou Zhu, Michael Angell, Chun-Jern Pan, Meng-Chang Lin, Hui Chen, Chen-Jui Huang, Jinuan Lin, Andreas J. Achazi, Payam Kaghazchi, Bing-Joe Hwang, Hongjie Dai

**Affiliations:** Department of Chemistry, Stanford University Stanford California 94305 USA hdai1@stanford.edu; Department of Chemical Engineering, National Taiwan University of Science and Technology Taipei 10607 Taiwan; College of Electrical Engineering and Automation, Shandong University of Science and Technology Qingdao 266590 People's Republic of China; Physikalische und Theoretische Chemie, Freie Universität Berlin Takustr. 3 D-14195 Berlin Germany; Forschungszentrum Jülich GmbH, Institute of Energy and Climate Research (IEK-1), Materials Synthesis and Processing Wilhelm-Johnen-Straße, 52425 Jülich Germany; Department of Chemistry, University of South Dakota 414 E. Clark St. Vermillion SD 57069 USA

## Abstract

Room temperature ionic liquids (RTILs) are solvent-free liquids comprised of densely packed cations and anions. The low vapor pressure and low flammability make ILs interesting for electrolytes in batteries. In this work, a new class of ionic liquids were formed for rechargeable aluminum/graphite battery electrolytes by mixing 1-methyl-1-propylpyrrolidinium chloride (Py13Cl) with various ratios of aluminum chloride (AlCl_3_) (AlCl_3_/Py13Cl molar ratio = 1.4 to 1.7). Fundamental properties of the ionic liquids, including density, viscosity, conductivity, anion concentrations and electrolyte ion percent were investigated and compared with the previously investigated 1-ethyl-3-methylimidazolium chloride (EMIC-AlCl_3_) ionic liquids. The results showed that the Py13Cl–AlCl_3_ ionic liquid exhibited lower density, higher viscosity and lower conductivity than its EMIC-AlCl_3_ counterpart. We devised a Raman scattering spectroscopy method probing ILs over a Si substrate, and by using the Si Raman scattering peak for normalization, we quantified speciation including AlCl_4_^−^, Al_2_Cl_7_^−^, and larger AlCl_3_ related species with the general formula (AlCl_3_)_*n*_ in different IL electrolytes. We found that larger (AlCl_3_)_*n*_ species existed only in the Py13Cl–AlCl_3_ system. We propose that the larger cationic size of Py13^+^ (142 Å^3^) *versus* EMI^+^ (118 Å^3^) dictated the differences in the chemical and physical properties of the two ionic liquids. Both ionic liquids were used as electrolytes for aluminum–graphite batteries, with the performances of batteries compared. The chloroaluminate anion-graphite charging capacity and cycling stability of the two batteries were similar. The Py13Cl–AlCl_3_ based battery showed a slightly larger overpotential than EMIC-AlCl_3_, leading to lower energy efficiency resulting from higher viscosity and lower conductivity. The results here provide fundamental insights into ionic liquid electrolyte design for optimal battery performance.

## Abstract 2

Room temperature ionic liquids (RTILs) are solvent-free liquids comprised of densely packed cations and anions. Properties of Py13Cl–AlCl_3_ ILs were studied and compared with EMIC-AlCl_3_ ILs for use as electrolyte in Al–graphite battery.

## Graphical abstract

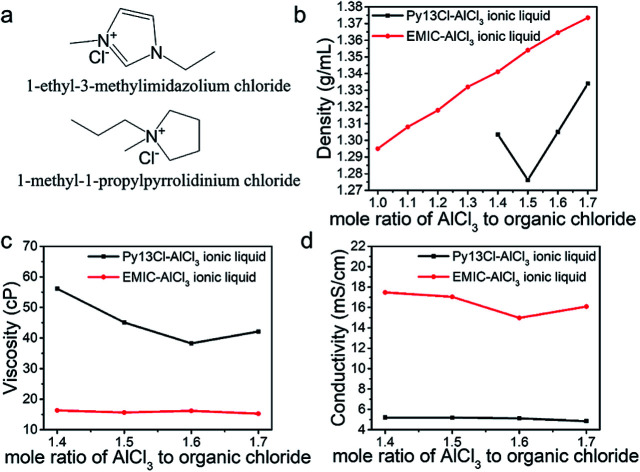

## Introduction

In recent years, with the increased deployment of portable devices, electric vehicles and renewable energy, rechargeable batteries with high energy density, power density, safety and long cycle life at low cost become highly desired. Lithium ion batteries (LIBs) have high energy density and high capacity and are regarded as one of the most promising energy storage devices. In addition to LIBs, other types of battery have been developed including sodium-ion batteries, zinc-ion batteries, magnesium-ion batteries and aluminum-ion batteries (AIBs) that could complement or serve as alternatives to each other.^1–9^

The electrolyte lies at the heart of a battery. With the advances in battery technology, the development of a safe and stable electrolyte is critically important. Room temperature ionic liquids (RTILs) are safe and sufficiently conducting, useful as battery electrolytes.^10–14^ Various ionic liquids have been investigated for different types of batteries, including LIB and AIB.^2,15,16^ Our group has developed rechargeable Al–graphite battery based on two types of electrolytes, an IL electrolyte made by mixing 1-ethyl-3-methylimidazolium chloride (EMIC) and AlCl_3_ and an quasi IL or deep-eutectic solvent (DES) by mixing urea with AlCl_3_.^7–9^ The batteries operate by reversible redox of Al at the negative Al foil electrode, and reversible carbon redox through chloroaluminate anion intercalation and de-intercalation at the graphite positive electrode.^7–9,17–19^ Still, much room exists in developing new IL electrolytes to improve Al battery, and especially, to understanding the relations between the composition, physical properties of IL electrolytes and battery performance.

Herein, we report a new series of ionic liquids formed by mixing 1-methyl-1-propylpyrrolidinium chloride and AlCl_3_ at various ratios (AlCl_3_/Py13Cl ratios: 1.4, 1.5, 1.6, 1.7). The electrolytes exhibited different physical and chemical properties compared to the widely used EMIC-AlCl_3_ ionic liquids. We devised an approach to probe and quantify the species in both ionic liquids containing monomeric AlCl_4_^−^ anion and dimeric Al_2_Cl_7_^−^ anion. We found that larger AlCl_3_ related species in the form of (AlCl_3_)_*n*_ existed only in Py13Cl–AlCl_3_ ionic liquid and were absent in EMIC-AlCl_3_. In addition, the overall concentration of AlCl_4_^−^ and Al_2_Cl_7_^−^ and ion percent were lower in the Py13Cl–AlCl_3_ system. The difference in cation size (Py13^+^: 142 Å^3^*versus* EMI^+^: 118 Å^3^) was likely responsible for the differences in the physical properties of Py13Cl–AlCl_3_ and EMIC-AlCl_3_ ILs. Batteries using Py13Cl–AlCl_3_ electrolyte showed lower energy and voltage efficiency as a result of their larger overpotential resulted from higher viscosity and lower ionic conductivity with the presence of large (AlCl_3_)_*n*_ species in the ionic liquid. Our results help to shed light into electrolyte design for Al batteries.

## Results

### Structure, density, viscosity, and conductivity of ILs

**Fig. 1:**
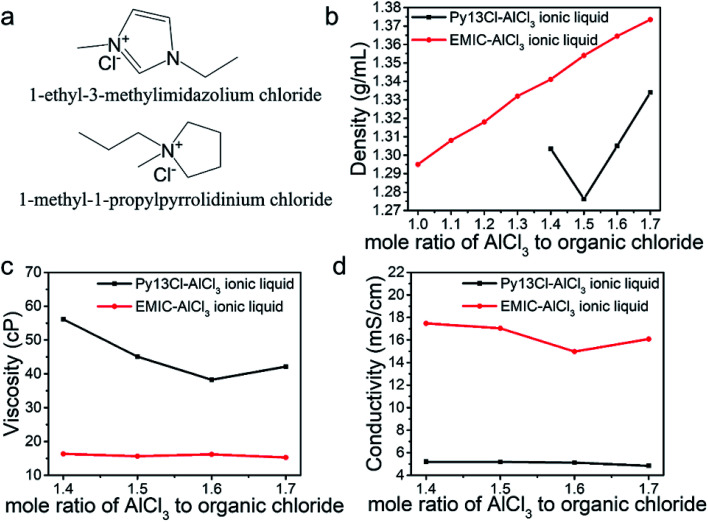
Structures and physical properties of Py13Cl–AlCl_3_ and EMIC-AlCl_3_ ionic liquid. (a) The structure of EMIC and Py13Cl, (b) density comparison between Py13Cl–AlCl_3_ and EMIC-AlCl_3_, (c) viscosity comparison between Py13Cl–AlCl_3_ and EMIC-AlCl_3_ measured at 23–24 °C. (d) Conductivity comparison between Py13Cl–AlCl_3_ and EMIC-AlCl_3_ measured at 25 °C.

Fig. 1a shows the structure of Py13Cl and EMIC. DFT calculations (B3LYP-D3BJ/def2-TZVP) were performed to determine the geometrically optimized structure and the electrostatic potential maps of Py13^+^, EMI^+^ and AlCl_4_^−^ (Fig. S1†). Subsequently the sizes of the molecules were determined based on the van der Waals radii to be 142 Å^3^, 118 Å^3^, and 105 Å^3^, respectively. AlCl_4_^−^ size ratio to Py13^+^ and EMI^+^ is 0.74 and 0.89, respectively.

We first measured the density of ionic liquids formed by mixing AlCl_3_ with Py13Cl and EMIC respectively at various molar ratios (Fig. 1b). The EMIC-AlCl_3_ ionic liquid density increased linearly with the AlCl_3_/EMIC ratio in the 1–1.7 range, in close agreement with literature reported results.^20^ A comparison between our experimental results and those calculated from literature was shown in Fig. S2† (temperature used for density calculation was 25 °C).^20^ A significant difference between the two ionic liquids was that well behaved liquids for the Py13Cl–AlCl_3_ system could not form for AlCl_3_/Py13Cl < 1.4, unlike the homogeneous clear liquids formed for AlCl_3_/EMIC ≥ 1. For the Py13Cl–AlCl_3_ system, a gel like mixture was formed with visible precipitates when AlCl_3_/Py13Cl = 1–1.3. Also different was that for AlCl_3_/Py13Cl > 1.3, the change in density of Py13Cl–AlCl_3_ ionic liquid did not follow a linear trend with the increase in AlCl_3_/Py13Cl molar ratio. Density decreased first from AlCl_3_/Py13Cl = 1.4 to 1.5 and then increased as AlCl_3_/Py13Cl further increased (Fig. 1b black curve).

We also measured viscosity of the two ionic liquid systems at temperature of 23 to 24 °C. The viscosity of Py13Cl–AlCl_3_ ionic liquid was about 3 times higher than that of EMIC-AlCl_3_ ionic liquid (Fig. 1c), with its viscosity decreased as the AlCl_3_/Py13Cl ratio changed from 1.4 to 1.6 and then slightly increased as the AlCl_3_ ratio further increased to 1.7. Conductivity measurements of these ionic liquids found that, corroborated with the higher viscosity of Py13Cl–AlCl_3_ ionic liquid, its ionic conductivity, measured at 25 °C, was about 3 times lower than that of EMIC-AlCl_3_ (Fig. 1d).

### Speciation of ionic liquids probed by Raman spectroscopy

**Fig. 2:**
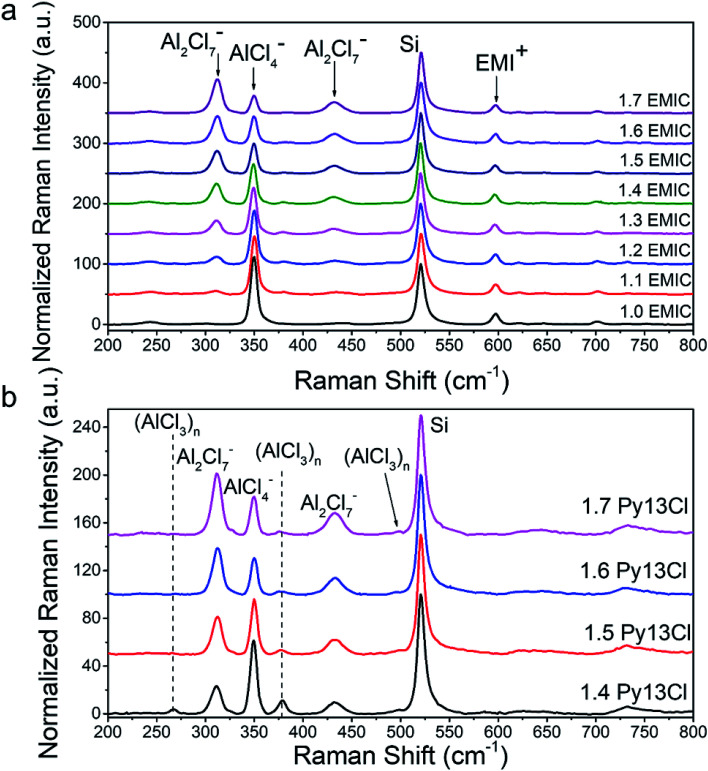
Raman spectra of Py13Cl–AlCl_3_ and EMIC-AlCl_3_ ionic liquid, normalized by the Si wafer peak at around 520 cm^−1^. (a) Raman spectra of EMIC-AlCl_3_ at different AlCl_3_ ratios, with species assignment to major peaks, (b) Raman spectra of Py13Cl–AlCl_3_ ionic liquid at different AlCl_3_ ratio, with species assignment to major peaks.

**Fig. 3:**
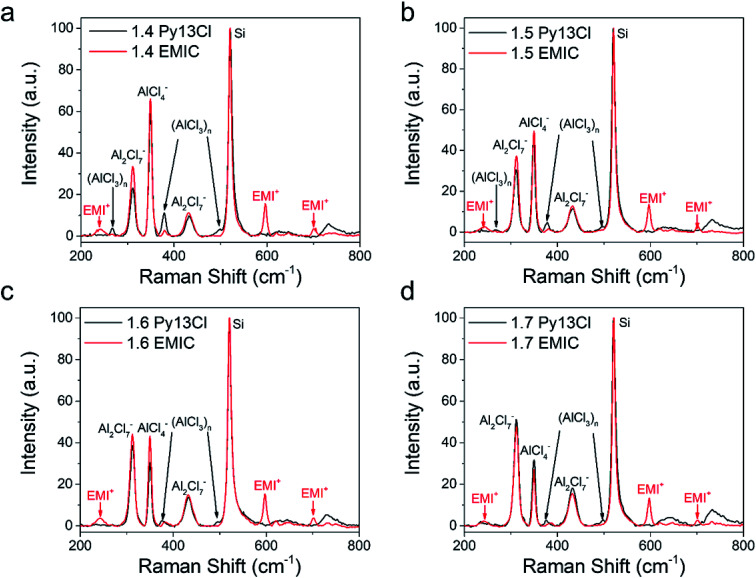
Raman spectra comparison between Py13Cl–AlCl_3_ and EMIC-AlCl_3_ ionic liquid. (a) 1.4 Py13Cl and 1.4 EMIC comparison, (b) 1.5 Py13Cl and 1.5 EMIC comparison, (c) 1.6 Py13Cl and 1.6 EMIC comparison, (d) 1.7 Py13Cl and 1.7 EMIC comparison.

Fig. 2a and b showed the Raman spectra of EMIC-AlCl_3_ and Py13Cl–AlCl_3_ ionic liquids, respectively. A piece of p-type boron doped silicon wafer was placed inside a clear plastic pouch containing the IL, and micro-Raman was done by focusing the laser through the clear plastic pouch onto the Si wafer surface to obtain spectra of both the Si and ILs within the laser focal volume. All spectra were taken when the silicon signal was maximized and all the peaks were then normalized to Si. The peaks at around 311 cm^−1^ and 433 cm^−1^ were known to belong to dimeric Al_2_Cl_7_^−^, and the peak at around 350 cm^−1^ was assigned to monomeric AlCl_4_^−^.^7,9,13,21,22^ The peak at around 520 cm^−1^ was the silicon wafer and normalized to 100. Small peaks at around 240 cm^−1^, 383 cm^−1^, 597 cm^−1^, 630 cm^−1^, 650 cm^−1^, 700 cm^−1^ all belonged to the EMI^+^ (Fig. 3). Some of them were also observed by Takahashi *et al.* and assigned to EMI^+^ in their study of EMIC-AlCl_3_ ionic liquid.^21^ In addition, the Raman spectrum of pure EMIC solid was taken and compared with the 1.7 EMIC IL, and the result further confirmed the validity of this peak assignment (Fig. S3†). The peaks at 311 cm^−1^ and 433 cm^−1^ increased in intensities and the peak at 350 cm^−1^ decreased in intensity as more AlCl_3_ was added, indicating that more Al_2_Cl_7_^−^ and fewer AlCl_4_^−^ were formed at higher AlCl_3_/EMIC or AlCl_3_/Py13Cl ratios. The chemical equations govern these reactions were as follows:^23–25^

1a AlCl_3_ + EMIC → EMI^+^ + AlCl_4_^−^ (AlCl_3_ ratio ≤ 1)

1b AlCl_3_ + Py13Cl → Py13^+^ + AlCl_4_^−^ (AlCl_3_ ratio ≤ 1)

1c AlCl_3_ + AlCl_4_^−^ → Al_2_Cl_7_^−^ (1 < AlC_3_ ratio < 2)

Three peaks unique to the Py13Cl–AlCl_3_ ionic liquids were observed at ∼270 cm^−1^, 377 cm^−1^ and 495 cm^−1^ (Fig. 3). These peaks were assigned to be neutral-like AlCl_3_ species in the form of aggregates, dimers, multimers and (AlCl_3_)_*n*_ species. Peaks near 280 cm^−1^ were assigned to neutral aluminum chloride in the literature depending on the experimental conditions and chemical environment.^26–28^ The peak at 377 cm^−1^ was assigned to Al_3_Cl_10_^−^ by Dymek *et al.* in their spectral study of Al_3_Cl_10_^−^, and the shoulder peak at 495 cm^−1^ was also observed by Rytter *et al.* in their Raman spectroscopic investigation of the melts of AlCl_3_ and AlkCl (Alk = Li, K, Cs).^26,29^ Peak at ∼495 cm^−1^ was present when AlCl_3_ concentration exceeded 66.7 mol% and the authors assigned it to higher polymeric Al_*x*_Cl_3*x*+1_^−^ ions, with the possibility of *x* > 3.^26^ The peak position was also likely to shift depending on the cation size.^26^ These peaks were also observed in the inhomogeneous 1.3 Py13Cl–AlCl_3_ mixture.

### Quantitative speciation and ‘ion percent’ of electrolytes

From Raman spectra, we estimated the concentrations of AlCl_4_^−^ and Al_2_Cl_7_^−^ in the ionic liquids by using the Si normalized Raman intensity of the peaks at 311 cm^−1^ (Al_2_Cl_7_^−^) and 350 cm^−1^ (AlCl_4_^−^) respectively. In the 1.0 AlCl_3_ : 1.0 EMIC ionic liquid, the only species present were AlCl_4_^−^ and EMI^+^, and the molar concentration of AlCl_4_^−^ in the 1.0 IL electrolyte equaled to that of AlCl_3_ (mole number of AlCl_3_ in the IL/molar volume of the IL). The concentration of AlCl_4_^−^ at other AlCl_3_ ratios (*x*) can be calculated using the following equation.2
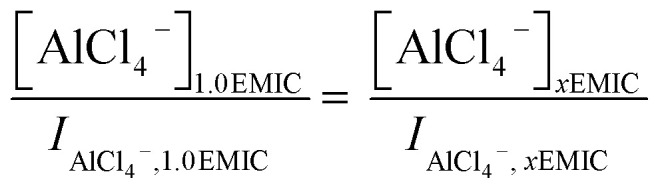


In eqn 2, *I* was the intensity of the AlCl_4_^−^ peak at 350 cm^−1^, and *x* was the molar ratio of AlCl_3_/EMIC ranging from 1.1 to 1.7. The dimeric anion concentration was calculated by3
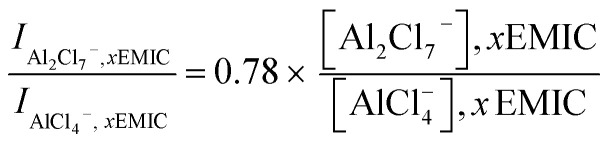
0.78 was the Raman cross section ratio between Al_2_Cl_7_^−^ and AlCl_4_^−^ in the EMIC-AlCl_3_ ionic liquid, determined from the method described by Gilbert *et al.*^30^

For the Py13Cl–AlCl_3_ ILs, quantitative analysis of the speciation was not as straightforward due to the inability in forming a AlCl_3_/Py13Cl = 1.0 ratio electrolyte. We analyzed the concentrations of AlCl_4_^−^ and Al_2_Cl_7_^−^ from their Raman peak intensities after normalizing the Raman spectra of the Py13Cl–AlCl_3_ and EMIC-AlCl_3_ electrolytes to the same Si reference placed into the two ionic liquids. By so doing we estimated the anions concentrations in the Py13Cl electrolytes through the normalized Raman intensities using4
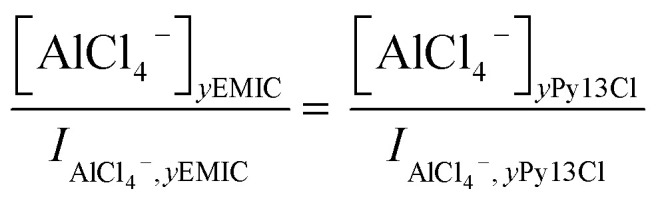
5
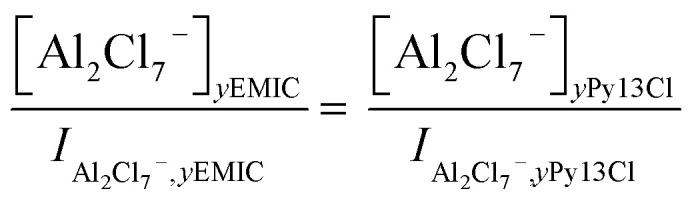


In eqn (4) and (5), *I* was the normalized intensity for AlCl_4_^−^ and Al_2_Cl_7_^−^ and *y* was the ratio of AlCl_3_ ranging from 1.4 to 1.7.

**Fig. 4:**
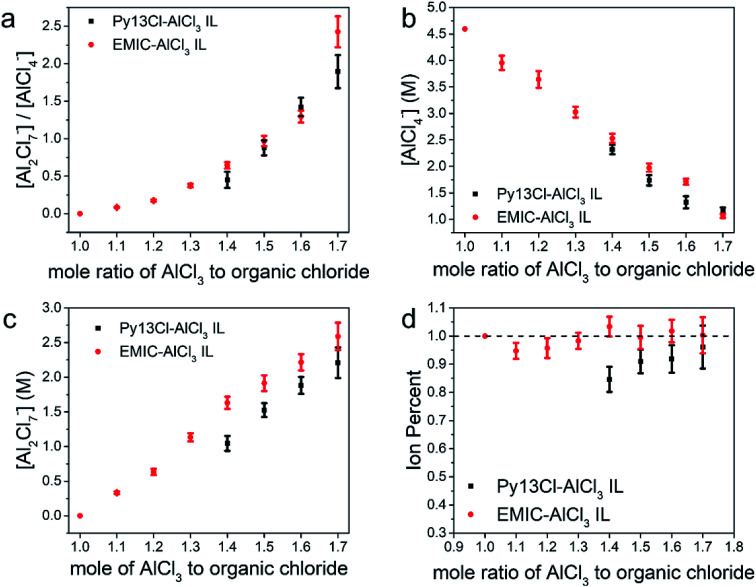
Species concentrations and ion percent comparison between Py13Cl–AlCl_3_ and EMIC-AlCl_3_ ionic liquids (a) [Dimer] to [Monomer] ratios comparison between these two ionic liquids, (b) monomer concentration comparison between these two ionic liquids, (c) dimer concentration comparison between these two ionic liquids, (d) ion percent comparison between these two ionic liquids.

The ratios between [Al_2_Cl_7_^−^] to [AlCl_4_^−^] were similar in both Py13Cl–AlCl_3_ and EMIC-AlCl_3_ ionic liquids, especially at AlCl_3_/organic chloride = 1.4–1.6 (Fig. 4a). In both systems, the monomeric anion concentration decreased with increasing AlCl_3_ ratio, and was lower in the Py13Cl–AlCl_3_ system than that in EMIC-AlCl_3_ at AlCl_3_ ratio equals to 1.4–1.6. When AlCl_3_/organic chloride = 1.7, the monomer anion concentration in both ionic liquids was similar (Fig. 4b). As expected, the Al_2_Cl_7_^−^ concentration increased as the AlCl_3_ ratio increased (Fig. 4c), and was always lower in the Py13Cl–AlCl_3_ IL than in the EMIC-AlCl_3_ IL (Fig. 4c). This made the overall concentrations of AlCl_4_^−^ and Al_2_Cl_7_^−^ lower in the Py13Cl–AlCl_3_ IL than that in the EMIC-AlCl_3_ IL at a given AlCl_3_ to organic chloride ratio (Fig. 4b and c).

We defined a term “ion percent” as the ratio between [AlCl_4_^−^] + 2 × [Al_2_Cl_7_^−^] and [AlCl_3_]. By so doing we only included [AlCl_4_^−^] and [Al_2_Cl_7_^−^] since they were the only electrochemically active species in our ILs for Al battery operation. If the ion percent was 1, it indicated that all AlCl_3_ were consumed for making monomers and dimers. When the ion percent was less than 1, larger (AlCl_3_)_*n*_ could form. For EMIC-AlCl_3_ IL, the ion percent values were near 1.0 (Fig. 4d), suggesting anions in the electrolytes were mostly in the form of AlCl_4_^−^ and Al_2_Cl_7_^−^. In the Py13Cl–AlCl_3_ system, however, this ion percent value was always lower. When the AlCl_3_ ratio to Py13Cl was 1.4 (the lowest required to form a liquid), the ion percent was at its lowest, 0.85, and increased slightly as more AlCl_3_ was added and was always lower than 1. This trend in ion percent was consistent with the observations of the three unique peaks (270 cm^−1^, 377 cm^−1^, 495 cm^−1^) in the Py13Cl–AlCl_3_ Raman spectra. As the AlCl_3_ content increased, all these peaks had their intensities decreased, with the peaks at 270 cm^−1^ and 377 cm^−1^ being the most obvious. This trend suggested reduced concentrations of (AlCl_3_)_*n*_ species as AlCl_3_/Py13Cl increased, which was also reflected by the slight increase in ion percent for the Py13Cl–AlCl_3_ IL. In the EMIC-AlCl_3_ spectra, however, these three peaks were absent, which was consistent with its ion percent value always close to 1. The error bars in Fig. 4 were obtained using formulas from error propagation (eqn S1†).

### Cyclic voltammetry and battery data

**Fig. 5:**
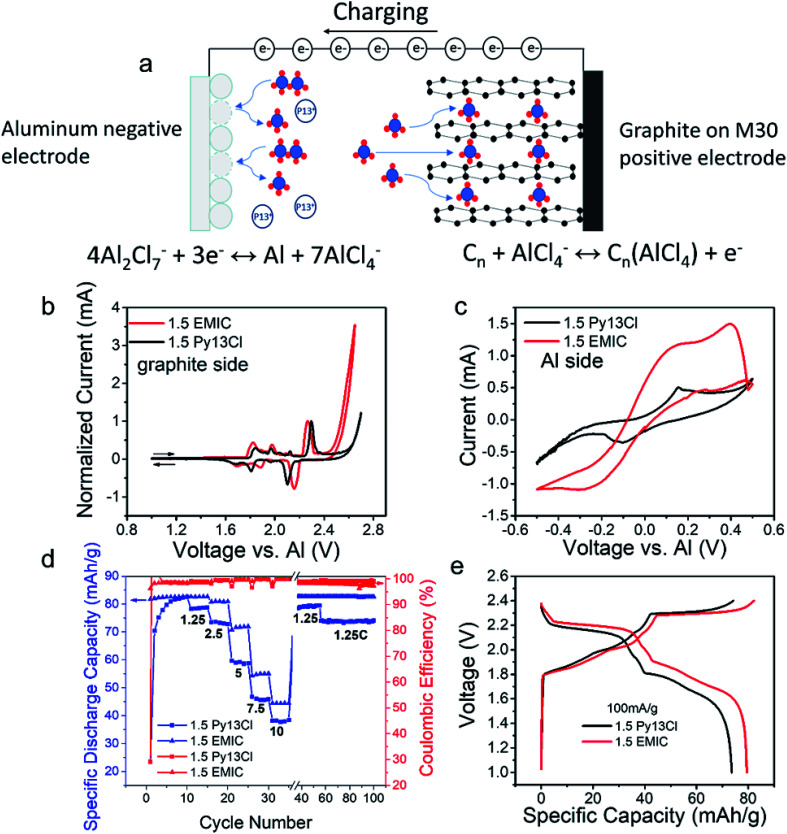
Aluminum–graphite battery performances when Py13Cl–AlCl_3_ and EMIC-AlCl_3_ ionic liquids were used as electrolyte (a) schematic depiction of how the Al–graphite battery worked, (b) cyclic voltammetry data at graphite electrode, three electrodes CV with Al as reference, (c) cyclic voltammetry data at Al electrode, three electrodes CV with Al as reference, (d) stability and capacity of Al–graphite batteries using 1.5 Py13Cl and 1.5 EMIC as electrolyte (C-rate were indicated in the figure. For 1.5 Py13Cl battery, cycle 1–10: cutoff voltage 2.6 V, cycle 11–55: cutoff voltage 2.5 V, cycle 56–100: cutoff voltage 2.4 V. For 1.5 EMIC battery, cutoff voltage was 2.4 V for all cycles), (e) Al battery charge–discharge curves comparison between 1.5 Py13Cl and 1.5 EMIC as electrolytes.

The Py13Cl–AlCl_3_ ionic liquid was used as an electrolyte for rechargeable aluminum–graphite battery (Fig. 5a). A simplistic battery operation mechanism was that during charging, AlCl_4_^−^ in the electrolyte intercalated into the positive electrode and oxidized the graphite, making C_*n*_(AlCl_4_^−^) compound with electrons released. At the negative electrode, Al_2_Cl_7_^−^ in the electrolyte was reduced to Al metal and formed AlCl_4_^−^ that migrated to the positive electrode side.^7,9^ When the battery was discharged, the opposite reactions occurred. At the negative electrode, aluminum metal was oxidized to Al_2_Cl_7_^−^ by consuming AlCl_4_^−^ in the electrolyte. At the positive electrode, AlCl_4_^−^ deintercalated from the graphite and reduced C_*n*_(AlCl_4_^−^) to C_*n*_.

Cyclic voltammetry of the graphite electrodes (Fig. 5b) and aluminum electrode (Fig. 5c) in Al batteries were performed in 1.5 AlCl_3_ : 1.0 EMIC and 1.5 AlCl_3_ : 1.0 Py13Cl electrolytes respectively (scan rate = 0.58 mV s^−1^ with an Al metal reference electrode). The overall shapes of these two curves were somewhat similar, but obvious difference was observed. The 1.5 AlCl_3_/Py13Cl electrolyte showed a slightly higher voltage window. The irreversible reaction did not appear until a potential of 2.6 V, whereas in the 1.5 AlCl_3_/EMIC electrolyte the irreversible reaction appeared at 2.4 V. The overpotential (voltage difference in redox peaks) in the Py13Cl based electrolyte was higher than that in the EMIC based electrolyte, attributed to higher parasitic resistance due to the higher viscosity and lower conductivity of the Py13Cl system. The graphite side CVs had current normalized because the graphite electrodes loading for the two CVs were too low to keep the mass exact (Experimental methods section). Aluminum redox was clearly observed in both systems (Fig. 5c). It was observed that at the same voltage, the 1.5 EMIC battery showed higher current density than those in 1.5 Py13Cl battery, suggesting more facile Al redox reaction in the EMIC based electrolyte. The aluminum side CVs didn't need normalization as the size of the aluminum electrodes in the two CVs were kept the same (Experimental methods section).

The aluminum–graphite battery using 1.5 AlCl_3_ : 1.0 Py13Cl as electrolyte showed activation behavior during initial cycling (Fig. 5d), after which clear discharge voltage plateaus at around ∼2.2 V and ∼1.8 V appeared (Fig. 5e black curve). The battery was then cycled at various current densities (100 mA g^−1^ to 800 mA g^−1^) to investigate the rate performance, with high coulombic efficiency in the range of 99% to 100%. The battery at 100 mA g^−1^ current under a cutoff voltage of 2.4 V showed a capacity around 75 mA h g^−1^ with a coulombic efficiency about 99.2%. The discharging energy could be maintained at around 141 mW h g^−1^ (based on the graphite mass) with an energy efficiency about ∼89%. The aluminum–graphite battery using 1.5 AlCl_3_ : 1.0 EMIC as electrolyte could operate from 1 V to 2.4 V and no activation was needed in the beginning. Both batteries had similar stability over 100 cycles of charge–discharge (Fig. 5d). Comparison of the charge–discharge curves between 1.5 Py13Cl and 1.5 EMIC batteries at a current density of 100 mA g^−1^ (Fig. 5e) showed a larger overpotential in the 1.5 Py13Cl based battery, consistent with the cyclic voltammetry data (Fig. 5b).

## Discussion

In this work, we investigated a new ionic liquid system based on Py13Cl and AlCl_3_ for rechargeable Al batteries. Although the battery performance failed to match that based on the commonly used EMIC and AlCl_3_ IL. The results led to fundamental insights into electrolyte composition, chemical and physical properties and their relation to battery performance.

We used Raman spectroscopy as a tool to probe and quantify chloroaluminate anionic species in different ionic liquids. In the EMIC-AlCl_3_ ILs, the peak at around 598 cm^−1^ assigned to be EMI^+^ was present in every spectrum. Therefore, besides the Si chip peak at around 520 cm^−1^, the EMI^+^ peak was also useful as an internal normalization factor to calculate AlCl_4_^−^ and Al_2_Cl_7_^−^ concentrations in EMIC-AlCl_3_ ILs for AlCl_3_/EMIC = 1.0–1.7. To this end, we first determined the concentration of EMI^+^ in every ratio of AlCl_3_ by the following equation.6
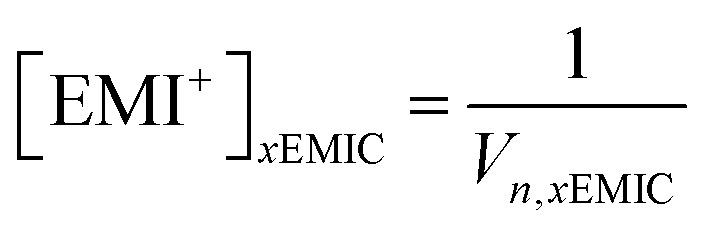


In eqn (6), *x* was the AlCl_3_ to EMIC ratio ranging from 1.0 to 1.7, and *V*_*n*_ was the molar volume of the IL, which could be determined from the average molecular weight dividing by the measured density. The EMI^+^ concentrations in different AlCl_3_ ratio ILs were different due to their difference in molar volume, originated from their difference in densities (Fig. 1b).

Next, the AlCl_4_^−^ and Al_2_Cl_7_^−^ intensity, normalized to EMI^+^, were calculated using the following equations.7
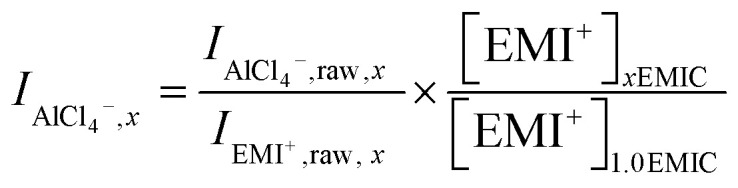
8
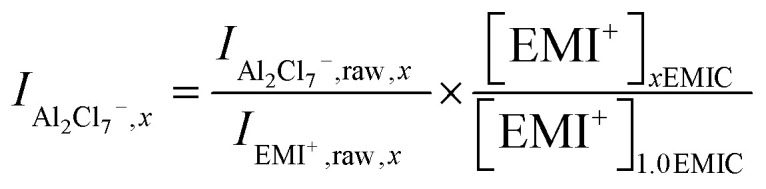


In eqn (7) and (8), subscript *x* was the ratio of AlCl_3_ to EMIC ranging from 1.0 to 1.7. *I*_AlCl_4_^−^,*x*_ and *I*_Al_2_Cl_7_^−^,*x*_ were the EMI^+^ normalized intensity for AlCl_4_^−^ and Al_2_Cl_7_^−^ in *x*EMIC, respectively. *I*_AlCl_4_^−^,raw,*x*_, *I*_Al_2_Cl_7_^−^,raw,*x*_, and *I*_EMI^+^,raw,*x*_ were the raw Raman intensity of AlCl_4_^−^, Al_2_Cl_7_^−^ and EMI^+^ in *x*EMIC. Lastly, [EMI^+^]_*x*EMIC_ and [EMI^+^]_1.0EMIC_ were the EMI^+^ concentration in *x*EMIC and 1.0 EMIC, calculated from eqn (6), respectively. The ratio of 
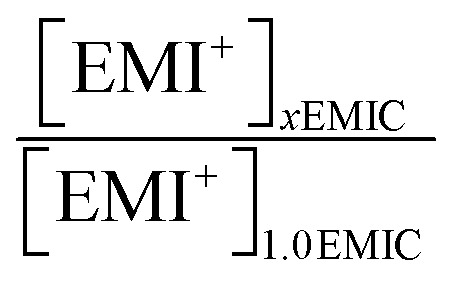
 was a correction factor for the EMI^+^ normalized intensity, due to the fact that EMI^+^ concentration were different in different AlCl_3_ ratio ILs.

After obtaining the EMI^+^ normalized peak intensity for AlCl_4_^−^ and Al_2_Cl_7_^−^ from eqn (7) and (8), these two quantities were plugged into eqn (2) and (3) to determine the AlCl_4_^−^ and Al_2_Cl_7_^−^ concentrations, similar to the Si normalization case. Ion percent could also be easily calculated using these newly obtained AlCl_4_^−^ and Al_2_Cl_7_^−^ concentrations. These results obtained by EMI^+^ normalization were compared with the Si normalization results (Fig. S4†), showing a high degree of agreement. This confirmed that the validity of the normalization method using Si as an external Raman reference. We believe that this method could be broadly applicable to facilitate quantitative anion speciation comparisons of a wide range ILs that lack a common cation Raman signature.

The Py13Cl–AlCl_3_ ionic liquids exhibited different properties (higher viscosity, lower conductivity, lower overall monomeric and dimeric anion concentrations and formation of large (AlCl_3_)*_n_* species) from the EMIC-AlCl_3_ ionic liquid, originated from the larger cationic size of Py13^+^ than the EMI^+^ cation (DFT calculated size of the Py13^+^ and EMI^+^ ∼142 Å^3^ and 118 Å^3^, respectively, Fig. S1†).^31^ When the cation size changed in an ionic liquid, it could greatly affect the chemical environment around it and its solvation shell. Bigger size cations could stabilize and favored the formation of larger species such as (AlCl_3_)_*n*_. In addition, the pi-system and the Brønsted acidic set of hydrogen atoms, which were unique in the EMI^+^ and absent in the Py13^+^, helped with solubilizing and liquidizing of the ionic liquid. Larger (AlCl_3_)_*n*_ species tend to form in the Py13Cl–AlCl_3_ system without forming a stable solvation shell.^32^ This trend was reported by several authors in the literature.^26,29^ Larger (AlCl_3_)_*n*_ species were only observed in Py13Cl–AlCl_3_ ionic liquid, and their concentration decreased as we increased the AlCl_3_ concentration.

We also calculated the interaction energy and the Gibbs free energy change for de-solvation in these two ILs (Table S1†). Our results showed that the interactions between EMI^+^ and AlCl_4_^−^ was always stronger than that between Py13^+^ and AlCl_4_^−^. Weaker interaction in the Py13Cl–AlCl_3_ electrolyte was mainly due to the larger size of Py13^+^, which decreased its effective positive charge and weakened its electrostatic interactions with AlCl_4_^−^. With a smaller interaction energy between Py13^+^ and AlCl_4_^−^, the equilibrium constant for eqn (1b), compared to eqn (1a), would be smaller. As a result, larger polymeric (AlCl_3_)_*n*_ species were present in some of the lower ratios Py13Cl IL. Once enough AlCl_3_ was introduced to the system, the total number of ions in the IL increased and these polymeric species started to disappear, as suggested by the diminishing of the Raman peak at 270 cm^−1^ (Fig. 3). In addition, unlike the homogeneous AlCl_3_/EMIC = 1 ionic liquid, this weaker electrostatic interaction made the formation of stable solvation shell in Py13Cl IL more difficult, which led to the inability of forming an IL for AlCl_3_/Py13Cl = 1. This phenomenon also suggested mismatch of cation and anion sizes at the cation/anion ratio = 1 condition to keep charge-neutrality while forming stable solvation shells with the same coordination numbers with counter-ion as in the AlCl_3_-EMIC case. When larger dimeric ions increased in concentration above a threshold level for AlCl_3_/Py13Cl ≥ 1.4 electrolytes, the system evolves into a well solvated liquid.

The Py13Cl–AlCl_3_ contained large species and lower overall concentrations of dimeric and monomeric anions. This combined with the larger size cations in the electrolyte afforded ILs exhibiting greater viscosity and lower ionic conductivity than the EMIC counterparts. This led to a larger overpotential for battery charge and discharge, giving lower energy and voltage efficiency as observed. In addition, the lower conductivity of this electrolyte also limited the current at the negative electrode, at which aluminum redox happened (Fig. 5c). Even though the cations in our electrolytes did not directly participate in any actual electrochemical reaction, they could affect the performance of the battery by controlling the anionic species around it, which in turn affected the physical properties of the IL including viscosity and conductivity. From our results, smaller cations could have positive effects on the battery, by decreasing the viscosity and increasing the conductivity of the resulting electrolyte. This could provide a guide to the synthesis of new ionic liquids for optimized batteries in the future.

## Conclusion

In this work, new ionic liquids were formed by mixing various ratios of AlCl_3_ with Py13Cl. The physical and chemical properties of resulting ionic liquid were investigated and they turned out to be very different from the commonly used EMIC-AlCl_3_ ionic liquid. At the same AlCl_3_/organic chloride ratio, Py13Cl–AlCl_3_ system had lower density, higher viscosity and lower conductivity than the EMIC-AlCl_3_ counterpart. Clear liquid could not form in Py13Cl–AlCl_3_ IL until AlCl_3_/Py13Cl molar ratio reached 1.4. Raman spectroscopy revealed monomeric AlCl_4_^−^ and dimeric Al_2_Cl_7_^−^ existed in both ILs, with their concentrations decreased and increased, respectively, as the content of AlCl_3_ was increased. The sum of [AlCl_4_^−^] and [Al_2_Cl_7_^−^] was lower in the Py13Cl–AlCl_3_ IL, in agreement with its lower conductivity. Large polymeric (AlCl_3_)_*n*_ species only existed in Py13Cl–AlCl_3_ IL. The properties for both ionic liquids as electrolytes in an aluminum–graphite battery were also compared. The batteries had similar capacity and similar stability. However, the battery with Py13Cl–AlCl_3_ as electrolyte had higher overpotential, which was due to its higher viscosity and lower conductivity. The cation/anion size in an IL can dictate its physical properties including density, viscosity and conductivity, and the battery performances such as overpotential, rate capabilities and energy efficiency. All of these are rooted in the solvation and coordination of ion-counter ions in the ionic liquid. Therefore, in order to synthesize better ionic liquids to be used as electrolyte, the cation size needs to be controlled carefully. Overall, RTILs are still very open for further investigation. With more and more discoveries and understanding on RTILs, their advantageous properties, including low flammability and high rate capabilities can be further utilized in energy storage.

## Conflicts of interest

There are no conflicts to declare.

## Supplementary Material

RA-009-C9RA00765B-s001
