# Supplementary material for: Rechargeable aluminum batteries: effects of cations in ionic liquid electrolytes
Source: RSC Adv. 2019 Apr 11;9(20):11322–30. doi: 10.1039/c9ra00765b (PMC9062991; doi:10.1039/c9ra00765b)
Supplement: RA-009-C9RA00765B-s001 [file RA-009-C9RA00765B-s001.pdf]

Electronic Supplementary Material (ESI) for RSC Advances.
This journal is © The Royal Society of Chemistry 2019

Supporting Information for

Rechargeable Aluminum Battery: Effects of Cations in Ionic Liquid Electrolytes

Guanzhou Zhu[1], Michael Angell[1], Chun-Jern Pan[1, 2], Meng-Chang Lin[3], Hui Chen[3], Chen-Jui Huang[2], Jinuan Lin[1], Andreas J. Achazi[4], Payam Kaghazchi[4, 5], Bing-Joe Hwang[2] and Hongjie Dai[1,*]

Experimental Methods

Synthesizing Py13Cl-$AlCl_3$ and EMIC-$AlCl_3$ ionic liquids. 1.5g of Py13Cl solid was added to a 20mL glass scintillation vial and was placed in a coffee cooler while the corresponding mass of $AlCl_3$ was weighing. (1.4 ratio: 1.711g, 1.5 ratio: 1.833g, 1.6 ratio: 1.955g, 1.7 ratio: 2.077g). The $AlCl_3$ was slowly added with constantly stirring. The mixture was transferred back and forth to the coffee cooler to keep the temperature low at all times. After adding all the $AlCl_3$, the mixture was left stirring until all the $AlCl_3$ dissolved. The same method was used to synthesize EMIC-$AlCl_3$ ionic liquid, just replacing the Py13Cl solid by EMIC solid.

Density, Viscosity, Conductivity measurement. The density of both the Py13Cl-$AlCl_3$ ionic liquids and EMIC-$AlCl_3$ ionic liquids was measured using a 2mL pycnometer (Thomas Scientific: 8350B16). The viscosity measurements were done by using NDJ-9S viscometer. The conductivity was measured using a conductivity meter (Mettler Toledo, FiveEasy Plus).

Raman Measurements. Fresh samples of electrolyte were transferred into a small clear plastic pouch inside a glovebox filled with argon gas. A small piece of p-type boron doped silicon wafer was also put into the pouch. The pouch was well sealed inside the glovebox before transferring out. Spectra was acquired using a 532nm Ar^+ laser. When we acquired the spectrum, we started with a continuous scan with scan frequency of every one second. The focus of the laser was adjusted until the maximum signal of the silicon wafer was reached. Afterwards, the spectrum was acquired. The same procedure was repeated at different silicon wafer locations for at least three times.

Electrodes Preparation. The graphite slurry was made using 87% by weight graphite powder (GP) from Ted Pella, INC. Carbon Powder SP-1 Natural Flake, 3% by weight Poly(vinyl alcohol) from Sigma-Aldrich, average molecular weight 30000-70000, and 10% by weight Poly(acrylic acid) from Polysciences, Inc. 25% aqueous solution with molecular weight ~50000. The solvent for the slurry was D.I. water. The slurry was stirred overnight and coated onto Mitsubishi carbon fiber paper (30 g/m^2). The coated electrode was baked in 120 ^{0}C vacuum oven for 2 hours, and was kept in 80 ^{0}C vacuum oven afterwards. The aluminum foil we used was from Alfa Aesar, 0.25 mm thick, annealed, 99% (metal basis), Si and Fe (combined) typically 1%.

Battery Preparation. The graphite and aluminum electrodes were both pasted onto carbon tape (Ted Pella, 16073) in an aluminum laminated pouch cell case (MTI, EQ-alf-100-210). 3-mm nickel tabs (MTI, EQ-PLiB-NTA3) were used as current collector. Glass fiber filter paper (Whatman GF/A) was used as separator. The cell was put into 80 ^{0}C vacuum oven overnight before transferring into an argon filled glovebox, in which electrolyte was put into the cell. The cell was then well sealed inside the glovebox before transferring out for electrochemical measurements.

Electrochemical Measurements. Cyclic voltammetry (CV) was performed on a potentiostat/galvanostat (model CHI 760D, CH Instruments). The graphite slurry used for cyclic voltammetry was usually 1000 times diluted then regular graphite slurry. This was to make the graphite loading very small so the CV peaks could be well discerned. One additional aluminum foil, usually 1-2 mm wide, was pasted onto carbon tape inside the pouch cell to be used as reference electrodes. For aluminum side CV, working, counter, reference electrodes were all aluminum, and the working electrode had size (0.2 cm × 0.2 cm) much smaller than that of the counter electrode (1 cm × 1 cm). The charge-discharge test was performed using device from Neware, BTS80, Version 17.

Rationale for using Si wafer intensity as an external reference to determine the anionic species concentrations

Since there was no cation peak in the Py13Cl-$AlCl_3$ spectrum, and clear liquid would not form at $AlCl_3$/Py13Cl ratio equaled to 1, we could only use the silicon wafer signal as the external reference. In addition, we assumed that the laser beam would excite the same volume of electrolyte each time. We believe that such assumptions were reasonable considering the fact that only an estimation on the concentration were needed, and consistent data were produced when Si peak and EMI^+ peak were used as reference separately. We noticed that once the Si signal was maximized, all the other peaks in the spectrum had intensities that could vary by around 10%. For each $AlCl_3$/organic chloride ratio IL, a minimum of three spectra were taken at different silicon wafer positions, and background subtraction to each spectrum was performed. The intensities of the silicon wafer were all normalized to 100, and the intensities for $AlCl_4^-$ and $Al_2Cl_7^-$ were obtained by taking the average intensity over all the spectra we acquired. In this way, the inherent error from the actual experiment could be minimized. The standard deviation obtained from all the spectra were used to determine the errors in the concentration and ion percent calculations.

Density Functional Theory (DFT) and *Ab Initio* Molecular Dynamics (AIMD) Calculations

To find minimum-energy structures we carried out DFT and AIMD calculations using projector-augmented plane-wave code VASP[1] with Perdew-Burke-Ernzerhof (PBE) functional[2] and D3 correction.[3,4] The EMI^+-$AlCl_4^-$ and $Py13^+$-$AlCl_4^-$ systems were modelled by 22Å×22Å×22Å unit cells in which at least 12 Å vacuum exist along *x, y, z* directions. We used a gamma *k*-point and a cutoff energy of 470 eV. The AIMD simulations were performed for 17 ps and 16 ps in the case of EMI^+-$AlCl_4^-$ and $Py13^+$-$AlCl_4^-$, respectively, considering the NVT ensemble with a Nosé-Hoover thermostat and T=300 K. For both cases, two different configurations were first considered and calculated using DFT, followed by AIMD simulation on the energetically more favourable structure. Afterwards, we carried out DFT calculation on 2 minimum energy structures in the AIMD simulation. By comparing total energies of structures from AIMD and DFT (before AIMD simulation) we determined the minimum energy structures of EMI^+-$AlCl_4^-$ and $Py13^+$-$AlCl_4^-$.

The interaction energy ΔE and the Gibbs free energy change for de-solvation ΔG were calculated as described in Ref. 5 using Gaussian and TURBOMOLE with B3LYP/def2-TZVP and B3LYP-D3(BJ)/def2-TZVP.[6-15] The Gibbs free energy change for de-solvation ΔG is determined at 298.15 K by including the temperature dependent rovibrational contributions with the help of statistical thermodynamics. The sizes of the molecules were determined by creating a molecular surface with the COSMO approach based on the unmodified van der Waals radii.[16,17]

1. Kresse, G.; Furthmüller, J., *Phys. Rev. B: Condens. Matter. Mater. Phys.*, **1996**, 54 ,11169-11186.
2. Perdew, J. P.; Burke, K.; Ernzerhof, M., *Phys. Rev. Lett.*, **1996**, 77, 3865-3868.
3. Grimme, S.; Antony, J.; Ehrlich, S.; Krieg, S., *J. Chem. Phys.* **2010**, 132, 154104-154119.
4. Grimme, S.; Ehrlich, S. and Goerigk, L., *J. Comp. Chem.* **2011**, 32(7), 1456-1465.
5. Okoshi, M.; Yamada, Y.; Yamada, A.; Nakai, H., *Journal of The Electrochemical Society*,2013 *160 (11),* A2160-A2165
6. Gaussian 16, Revision A.03, Frisch, M. J. et al., Gaussian, Inc., Wallingford CT, 2016.
7. TURBOMOLE V7.0.1, A development of University of Karlsruhe and Forschungszentrum Karlsruhe GmbH, 1989–2007, TURBOMOLE GmbH, since 2007, 2015. Available from: http://www.turbomole.com
8. Ahlrichs, R.; Bär, M.; Häser, M.; Horn, H.; Kölmel, C.; Chem. Phys. Lett. 1989, *162(3)*, 165-169.
9. Dirac, P. A. M., *Proceedings of the Royal Society A* **1929**, *123*, 714–733.
10. Slater, J. C., *Physical Review* **1951**, *81(3)*, 385–390.
11. Vosko, S. H.; Wilk, L.; Nusair, M., *Canadian Journal of Physics* **1980**, *58(8)*, 1200–1211.
12. Lee, C.; Yang, W.; Parr, R. G., *Physical Review B* **1988**, *37(2)*, 785–789.
13. Becke, A. D., *The Journal of Chemical Physics* **1993**, *98(7)*, 5648–5652.
14. Weigend, F.; Häser, M.; Patzelt, H.; Ahlrichs, R., *Chem. Phys. Lett.* **1998**, *294*,143-152.
15. Weigend, F.; Ahlrichs, R., *Phys. Chem. Chem. Phys.* **2005**, *7*, 3297-3305.
16.Klamt, A.; Schüürmann, G., *Journal of the Chemical Society*, *Perkin Transactions* 2 **1993**, 5, 799–805.
17. Schäfer, A.; Klamt, A.; Sattel, D.; Lohrenz, J. C. W.; Eckert, F., *Physical Chemistry Chemical Physics* **2000**, 2, 2187-2193.

Error propagation formula for Figure 4

As an example, the error in [$AlCl_4^-$] (Figure 4b) was calculated as:

$$\sqrt{\left(\frac{\Delta[AlCl_4^-]_{1.0}}{[AlCl_4^-]_{1.0}}\right)^2+\left(\frac{\Delta I,[AlCl_4^-]_{1.0}}{I,[AlCl_4^-]_{1.0}}\right)^2+\left(\frac{\Delta I,[AlCl_4^-]_x}{I,[AlCl_4^-]_x}\right)^2}\times[AlCl_4^-]_x \quad \text{(S1)}$$

In equation S1, $\Delta[AlCl_4^-]_{1.0}$ was 0 (error in $AlCl_4^-$ concentration in 1.0 EMIC), $\Delta I,[AlCl_4^-]_{1.0}$ and $I,[AlCl_4^-]_{1.0}$ were the error and absolute value of the $AlCl_4^-$ Si normalized Raman peak intensity in 1.0 EMIC, respectively. $\Delta I,[AlCl_4^-]_x$ and $I,[AlCl_4^-]_x$ were the error and absolute value of the $AlCl_4^-$ Si normalized Raman peak intensity in x EMIC, where x = 1.1 – 1.7. $[AlCl_4^-]_x$ was the $AlCl_4^-$ concentration in x EMIC (x = 1.1 – 1.7) obtained from equation 2. The errors for other quantities, [$Al_2Cl_7^-$], [$Al_2Cl_7^-$] / [$AlCl_4^-$] and ion percent could be obtained similarly.

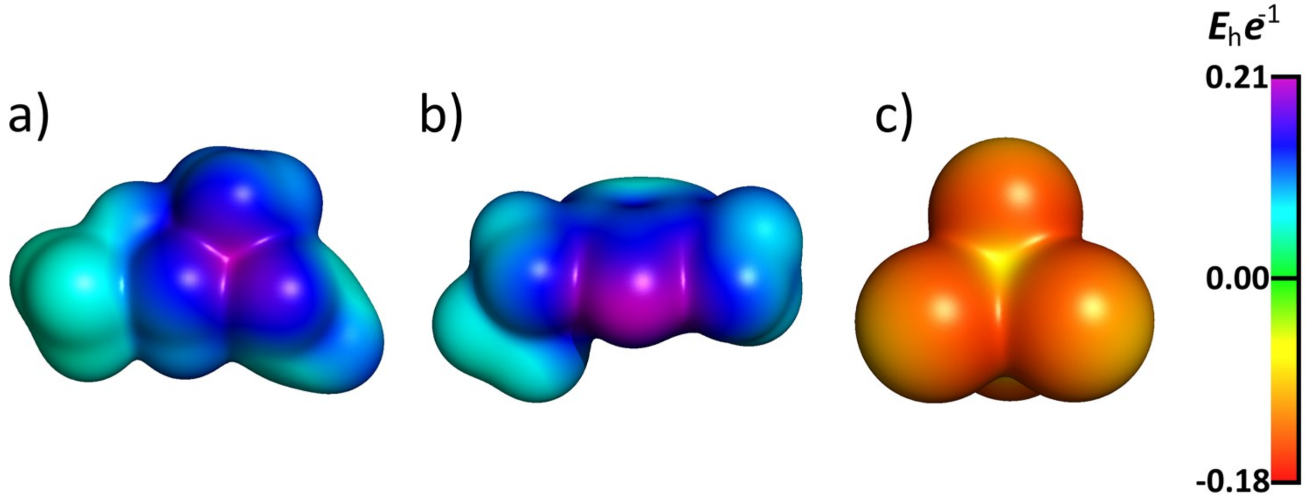

Figure S1 Electrostatic potential maps of a) $Py13^+$, b) EMI^+, and c)$AlCl_4^-$. Calculations were performed with B3LYP-D3(BJ). Red/orange indicates a negative and blue/purple a positive surface charge. The values in the scale bar are in E_h $\boldsymbol{e}^{-1}$ (Hartree per elementary charge). The sizes of the molecules were determined based on the van der Waals radii to be a) 142 $Å^3$, b) 118 $Å^3$, and c) 105 $Å^3$.

Ionic Liquid	Functional	De-solvation ΔG (kJ/mol)	Interaction Energy ΔE (kJ/mol)
EMIC-$AlCl_3$	B3LYP/def2-TZVP	249.41	304.25
	B3LYP-D3(BJ)/def2-TZVP	282.90	343.13
Py13-$AlCl_3$	B3LYP/def2-TZVP	243.93	297.31
	B3LYP-D3(BJ)/def2-TZVP	275.92	334.58

Table S1 De-solvation Gibbs free energy change (at 298.15 K) and interaction energy between EMI^+ and $AlCl_4^-$ as well as $Py13^+$ and $AlCl_4^-$ in EMIC-$AlCl_3$ IL and Py13Cl-$AlCl_3$ IL, respectively, calculated using B3LYP/def2-TZVP or B3LYP-D3(BJ)/def2-TZVP.

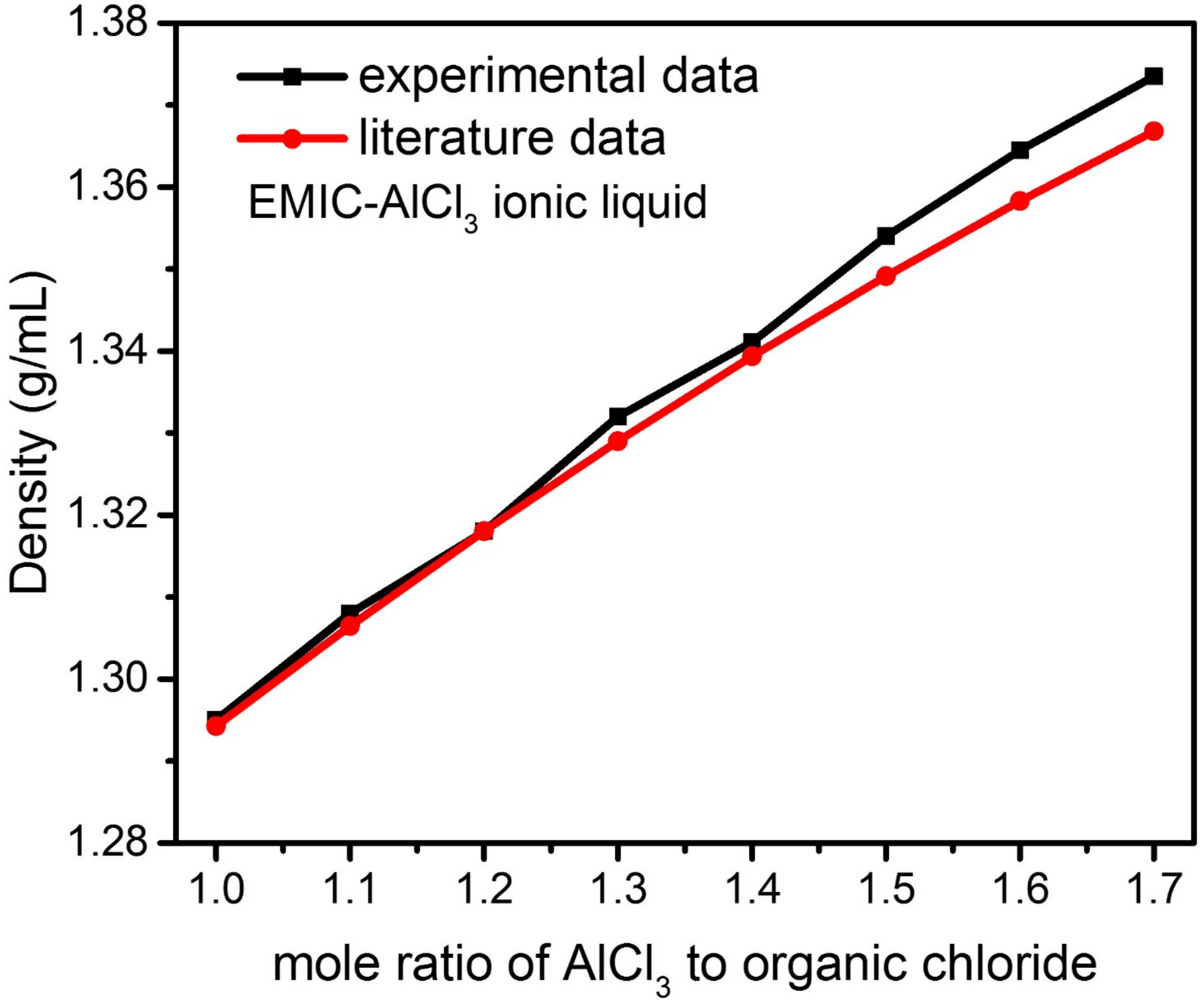

Figure S2 Density comparison between our experimental results and those obtained from literature.[20]

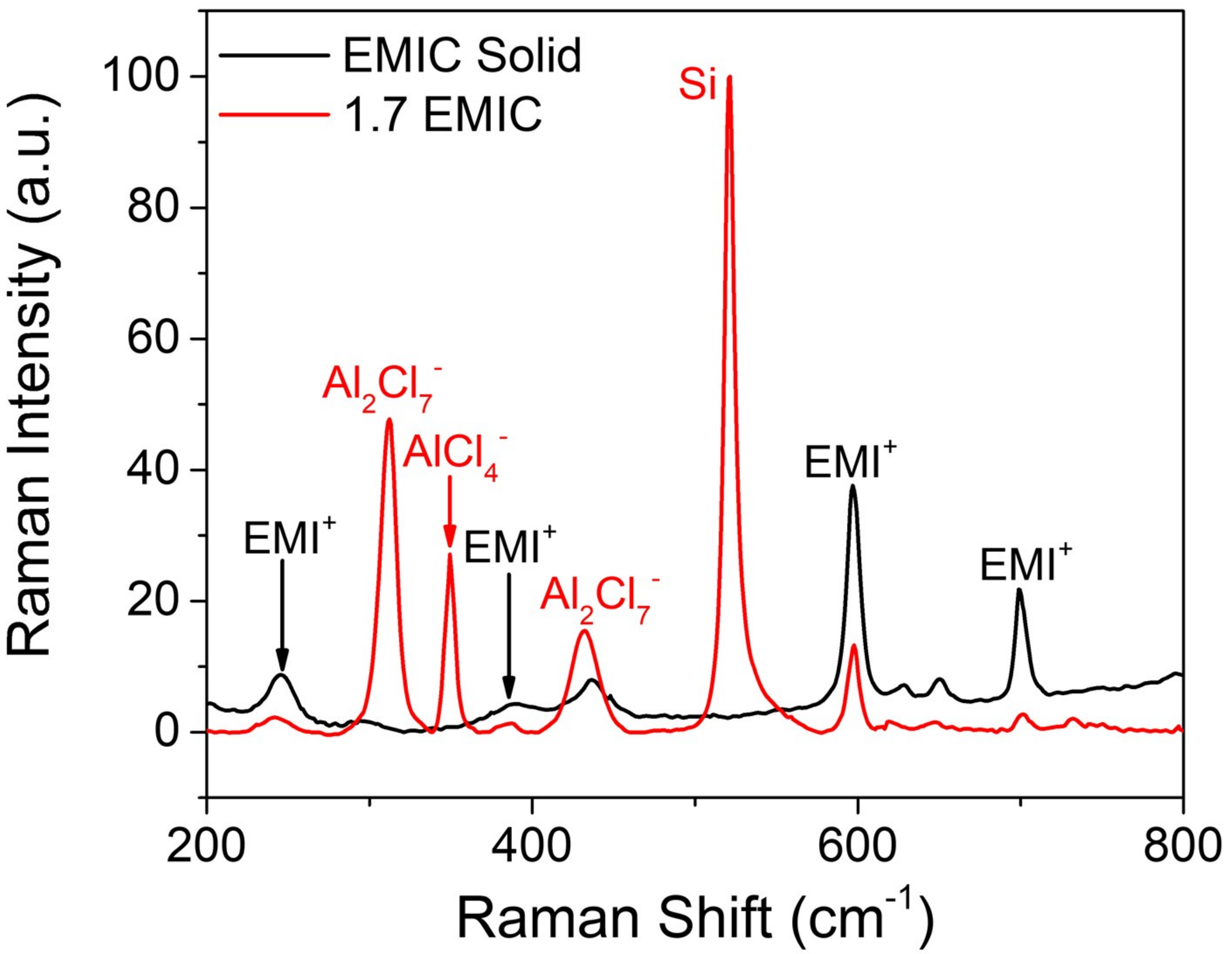

Figure S3 Raman spectrum comparison between EMIC solid and 1.7 EMIC ionic liquid.

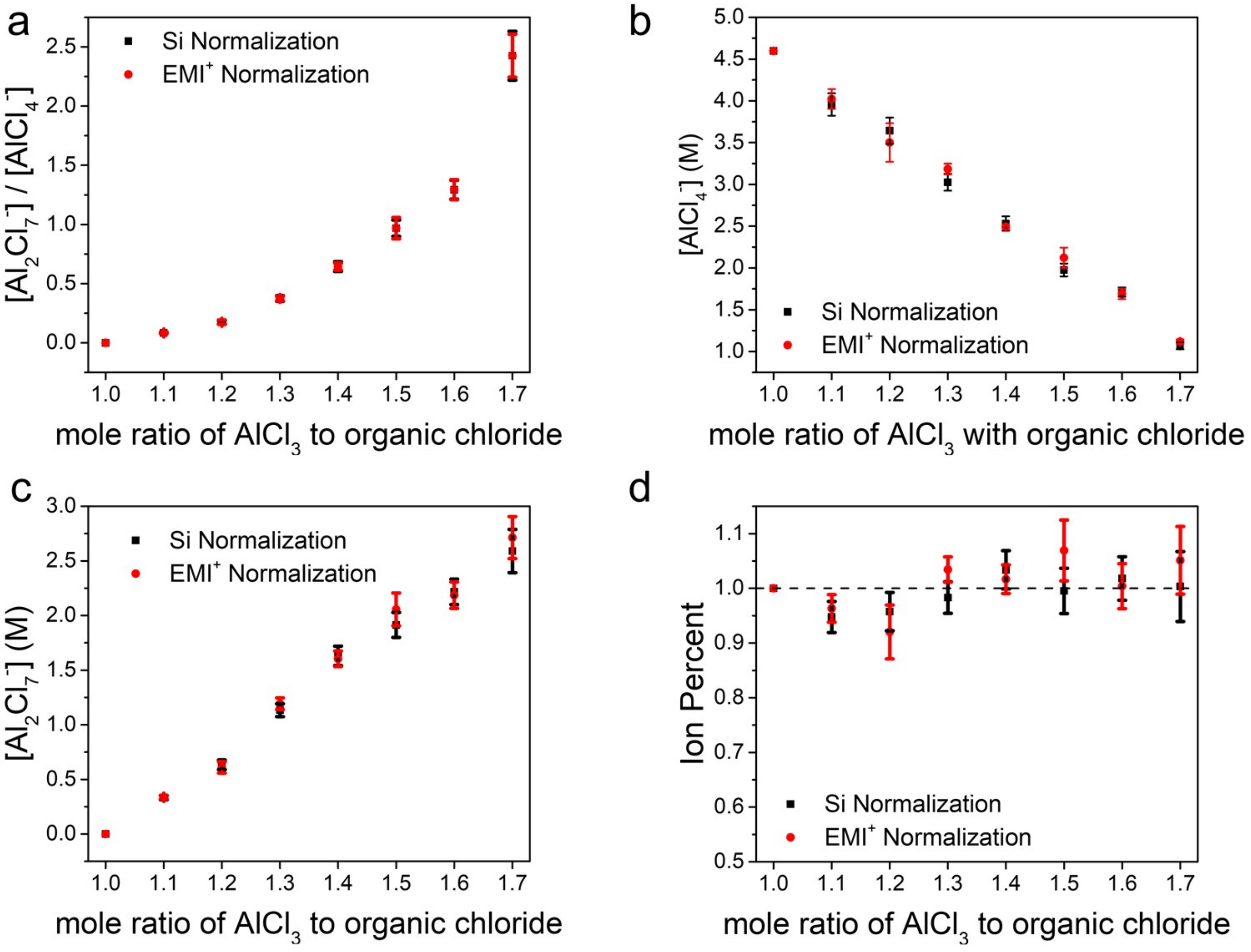

Figure S4 Comparison between EMI$^+$ normalization and Si normalization for $AlCl_4^-$, $Al_2Cl_7^-$ concentration and ion percent calculations. **a** comparison between the ratio of [$Al_2Cl_7^-$] and [$AlCl_4^-$] **b** comparison between the $Al_2Cl_7^-$ concentration **c** comparison between the $AlCl_4^-$ concentration **d** comparison between ion percent.
